# Can pilot free trade zones policy force the green transformation of enterprises? Evidence from listed companies in China

**DOI:** 10.1371/journal.pone.0301393

**Published:** 2024-05-30

**Authors:** Gulinaer Yusufu, Zhi Lu

**Affiliations:** School of Economics and Management, Xinjiang University, Urumqi, China; Inner Mongolia University, CHINA

## Abstract

In order to reveal the impact of pilot free trade zones policy on green development, we use multi-period difference-in-difference estimation and fixed effect model to explore the impact and impact mechanism of the establishment of free trade zones on the green transformation of enterprises from the micro perspective, based on the panel data of China’s A-share listed companies from 2009–2021, The results show that pilot free trade zones policy significantly improves the green transformation of enterprises in the zones. Pilot free trade zones policy affects the corporate green transformation through industrial agglomeration and financial constraints. The green transformation of state-owned enterprises, non-heavy polluting enterprises and high-tech enterprises are significantly impacted by pilot free trade zones policy. Urban innovation and green subsidies play a positive moderating role in the impact of free trade zones on enterprises’ green transformation. The research conclusions provide a valuable policy basis for how to promote the green transformation of enterprises under the free trade zones policy.

## 1. Introduction

The global economy is currently facing strong downturn, and the carbon emission reduction is becoming increasingly urgent for all the countries. According to the carbon emission figures published by the World Energy Statistics Yearbook 2021, China’s total carbon emissions in 2020 increased by 1.07 billion tons relative to 2011, an increase far higher than that observed for most countries [1]. The production of enterprises is the pillar and engine of China’s economic development, as well as the main source of energy consumption, environmental pollution and carbon emissions. Under this background, promoting the green transformation of enterprises has become the important way for China to implement the requirements of green development. For OPEC countries, policies aimed at reducing carbon emissions may not necessarily focus on limiting industrialization or energy consumption, which also implies that there may not be a need for immediate policy intervention to reduce carbon emissions through the regulation of energy consumption and industrialization. But for highly industrialised countries, there is a need for policy measures to reduce carbon emissions resulting from industrialization and energy consumption, particularly in the short term [2]. To realize a harmonious cohabitation between humans and nature, it is imperative to pursue green transformation and prioritize low-carbon growth as an essential approach. China, as a conscientious and influential nation, has declared its intention to reach the apex of carbon emissions by the year 2030 and attain carbon neutrality by 2060, thereby establishing a "dual carbon" target. Under the "dual carbon" target, carbon emission right is crucial asset for enterprises with carbon emission during the production process [3], which means enterprises can achieve a certain amount of carbon emissions for production. Without a doubt, enterprises that successfully undergo green transformation and development play a crucial role as significant micro market entities in accomplishing the "dual carbon" targets [4]. Nevertheless, the conflict between the objective of increasing profits for enterprises and the financial limitations, scarcity of skilled personnel, and restricted resources poses a persistent challenge to the implementation of environmentally-friendly initiatives. The topic of how enterprises can effectively achieve green transformation in alignment with the "dual carbon" agenda has emerged as a pressing concern that requires immediate resolution. Since its establishment in October 2013, the China (Shanghai) pilot free trade zone, henceforth referred to as the "Free Trade Zone," has experienced significant growth, expanding to 22 provinces, and spans from coastal to central and ultimately to western regions. Free trade zones play a crucial role in enhancing regional trade and investment by optimizing and adjusting institutional frameworks. This, in turn, fosters a more favorable economic climate for enterprises to undergo the green revolution.

We have systematically analyzed and summarized the previous research related to free trade zones. Research methodologies can be categorized into several approaches, including regression control method, multi-period dual analysis method, counterfactual analysis method, and composite control method. Some scholars used regression control method to examined the effects of establishing free trade zones on various economic indicators in the provinces of Shaanxi, Sichuan, and Chongqing, where existed notable variations in the influence of zones. Scholars also adopted a multi-period dual analytic approach to ascertain the substantial impact of pilot free trade zones policy on the high-quality development of non-state-owned enterprises. The effects of pilot free trade zones policy on regional economic development and innovation ability was positive [5]. Some scholars created counterfactual samples using the synthetic control approach in order to accomplish this goal [6]. The application of artificial control methods often demonstrates a propensity to overly focus on the study topic, sometimes ignores some important factors that influence the development of free trade zone. Although the composite control strategy appears to be a simple optimization issue, it may not work well if the variable value is beyond the control variable’s convex hull. An examination of counterfactual analysis was revealed a notable and favorable impact on Shanghai’s economic growth as a result of the implementation of the Shanghai Free Trade Zone [7]. It is evident that the policy implementation of pilot free trade zones has a notable and favorable impact on the advancement of regional economy in terms of high-quality development [8]. Pilot free trade zones policy has a noteworthy impact on both regional economic development and investment in innovation [6], and has the potential to facilitate equitable regional economic growth and foster the advancement of regional industries [9]. The establishment of free trade zones has played a significant role in facilitating the opening of factor markets, enhancing the efficient allocation of resources, fostering economic growth, boosting total factor productivity, and facilitating the advancement of high-quality economic development [10]. Moreover, Financial constraints have important impact on an enterprise’s research and development endeavors [11]. It has been seen that free trade zones exert a substantial and favorable influence on the overall amount of imports and exports [12].

Scholars examine the concept of "green transformation" through the prisms of entrepreneurship, industry, and the national economy. The green transformation at the national economic level entails a fundamental change in the structure of economic growth. This change involves the integration of both top-down and bottom-up methods and multi-level governance strategies, all aimed at attaining sustainable development objective [13]. The concept of green transformation encompasses a comprehensive restructuring process of multiple levels, including objective, system, demand, distribution, region, industry, input, and product [14]. The implementation of green transformation at the industrial level is perceived as a mutually beneficial scenario for attaining substantial industrial expansion and mitigating carbon emissions [15]. This approach aims to augment the role of green transformation in fostering economic growth within the industrial sector, while considering specific limitations related to energy consumption and environmental considerations. Some scholars underscored the significance of incorporating a range of factors, including human, financial, technological, policy, and environment in examinating the green transformation within the manufacturing industry [16]. This comprehensive approach aims to foster sustainable economic development in the manufacturing sector while also prioritizing environmental considerations. At the same time, the manufacturing industry is making full use of digital technology to promote industrial technological innovation and change of modes, so as to stimulate new momentum. Recently, many Chinese manufacturing enterprises have widely used digital technologies including artificial intelligence, big data, cloud computing and block chain, as well as digital management systems such as ERP and MES, to solve the problems of production and operation [17]. Under the change of development modes, it is important to consider various perspectives and approaches. The green transformation of enterprises is widely regarded as a means of attaining sustainable development or economic green development [18]. This transformation can be attributed to structural modifications resulting from energy innovation [19] or as an inevitable consequence of transitioning from a traditional economy to a green economy [20]. From the standpoint of green innovation, it is widely accepted among scholars that the fundamental aspect of corporate green transformation lies in green innovation, which encompasses various dimensions of innovation, such as green technology innovation [21], green product innovation, green management innovation, and green culture innovation [22]. Enterprises have the potential to enhance resource utilization and accomplish energy conservation and emission reduction objectives by implementing green innovation, which can lead to the realization of green transformation and sustainable development within enterprises [23]. The fundamental aspect of strategic change in enterprises is in the adoption of green development principles, green culture, and green strategies in the pursuit of environmental sustainability [24].

Scholars have initially examined the impact of formal environmental regulations, where the government assumes a central role in facilitating green transformation within enterprises, which includes government administrative orders [25] as well as administrative penalties and other mechanisms [26]. Moreover, a considerable body of research has been conducted to examine the effects of government environmental subsidies, environmental taxes, and pollution trading on company environmental sustainability initiatives [27]. Furthermore, environmental non-governmental organizations play a crucial role in facilitating the sustainable growth of enterprises [28]. Communities often bear the brunt of environmental pollution, making their role in compelling polluters to adopt environmentally friendly practices a crucial aspect that cannot be overlooked [29]. The growing environmental consciousness among the general population, along with their voluntary engagement in environmental practices and the subsequent media coverage, exerts a substantial influence on the adoption of sustainable practices by businesses [30]. The green transformation of enterprises is influenced by various factors such as resource allocation ability, green dynamic ability, innovation ability, strategic decision-making choice, strategic decision-making ability, energy structure, property rights organization form, enterprise heterogeneity, and executive heterogeneity etc. These factors are analyzed and discussed based on the resource-based theory, resource dependence theory, and heterogeneity theory of enterprises [31]. In the present circumstances of China’s ongoing pursuit of "carbon neutrality," enterprises, as significant participants in advancing the dual carbon objectives, assume a major role in facilitating the transition towards sustainability. Free trade zones grant a degree of independence in implementing reforms, improving the structure for global trade, and undertaking policy trials in several areas such as financial services, logistics services, commercial services, cultural services, and social services. Entry barriers such as qualification standards, equity limitations, and company scope constraints should be eliminated or suspended, specifically for international investors in designated regions, so that significantly improve market competitiveness. In addition, the advantages of free trade zones can also be seen in the independence of tax policies and tax administration. This implies that businesses within the zone can avail themselves of flexible taxation policies and supervision, resulting in a reduction of institutional transaction costs encountered by export-oriented enterprises during their operational activities [32]. The development of green finance positively affects enterprises’ sustainable green innovation. Moreover, enterprises’ financing restrictions and debt default risk play moderating roles, influence the relationship between green financing growth and green innovation. Enterprises’ continuous R&D investment is found to mediate this relationship [33]. Furthermore, considering the aspect of factor flow, the implementation of free trade zones has effectively eradicated obstacles to the capital flow, skilled labor, technology, and other factors across borders. This has facilitated the concentration of superior material resources and intellectual capital in specific regions, resulting in the formation of industrial clusters. Consequently, these developments have fostered a favorable environment for the adoption of environmentally sustainable practices by enterprises. The process of achieving a green transformation in organizations is characterized by its long-term nature and inherent uncertainty. To successfully implementing such a transformation, it is crucial to engage in continuous technical innovation activities and provide a consistent supply of factors. The implementation of free trade zones has facilitated the effectiveness of the movement of goods and personnel. Additionally, the inclusion of technological components has enhanced the potential for knowledge transfer, thereby established a framework for enterprises to pursue technological advancements and transition towards environmentally sustainable practices. Compared with previous studies, our research has following contributions: (1) Few studies have been conducted on the relationship between free trade zone and enterprise green transformation, our research can make up for the gap. (2) Previous studies only focus on the impact of pilot free trade zone policy on regional economy, our research focuses on the impact of free trade zone on micro-entity enterprises’ green transformation. (3) Our research enriches the previous studies by using the multi-period difference-in-differences method and by selecting enterprise Size (Size), monthly average excess turnover rate (Dturn), management expense growth rate (Managexpenratio), sales expense growth rate (Salesgrowth), liquidity Ratio (Liquidratio) and quick Ratio (Speedratio) as the control variables. (4) There is no previous research about the impact mechanism of the free trade zone on the enterprises’ green transformation, our research analyses the impact mechanism from the aspects of industrial agglomeration and financial constraints.

The structure of the remainder of this paper is as follows. In part two, we conduct theoretical analysis and research hypothesis. In part three, we conduct model construction and variable description. Then, in the part four, we conduct empirical analysis, including baseline regression estimation, parallel trend test and robustness test. In part five, we further analyse the impact of urban innovation and green subsidy. In the last part, we give the conclusions, summarize the limitations and future research planning.

## 2. Theoretical analysis and research hypothesis

Both advanced technology diffusion and independent innovation are crucial channels for latecon economies to achieve technological progress, based on the theory of economic development. So, pilot free trade zones policy can facilitate the advancement of green technology through the competition effect (which forces enterprises to innovate independently) and the technology spillover effect (which involves the diffusion, digestion, and absorption of technologies). The technology spillover theory reveals the transmission of advancements from an organization to other organizations. It has been demonstrated that negative listings and management models for foreign investment access within free trade zones are effective in facilitating investment and attracting a significant influx of enterprises. The entry of multinational corporations into the free trade zones facilitates the introduction of advanced technological research and development trends, as well as sophisticated green and clean technologies [34], which is achieved through the process of reverse knowledge spillover and demonstrative effects. Simultaneously, local enterprises stand to gain from the technology spillover effects of foreign-funded enterprises, which can potentially bridge the existing technological gap, which has the potential to enhance the innovative capacities and technological prowess of local enterprises. Enterprises in host country are able to attain greater levels of technical innovation through particular types of foreign investment liberalization, such as foreign direct investment (FDI), which is predicated on the practice of technological imitation [35]. In free trade zones, the further opening is conducive to attracting a large amount of foreign capital, which makes foreign direct investment bring demonstration effect, training and correlation effect in the industry. Promoting the gradual diffusion and transfer of green production technology of enterprises in the host country, realizing the combination of technological progress and green development is conducive to the realization of green technology progress of enterprises, and lays a foundation for the green transformation of enterprises. It can be seen that pilot free trade zones policy can promote the green transformation of enterprises through the entry of foreign investments. According to Porter’s "competitive strategy" theory, a favorable institutional environment in free trade zones is expected to attract a lot of multinational enterprises. Consequently, this influx is likely to result in a reduction of market share for existing enterprises and intensify the competition among them. Enterprises are motivated by the objective of maximizing profits, necessitating a constant enhancement of production efficiency through technical innovation to secure a larger market share and achieve higher profit margins. Therefore, the strengthening of foreign investment liberalization will intensify competition with domestic enterprises, thereby establishing a market selection mechanism characterized by the dominant position of the most adaptable entities [35]. Additionally, the establishment of free trade zones will facilitate the availability of diverse goods and services [36]. The substitutability between these goods and services will encourage local enterprises to increase their investment in R&D and gain market share through innovation. In the process of this investment, enterprises’ imitation of green technology causes certain competition effect and catch-up effect in the industry, thus promoting the development of green technology. Therefore, we propose hypothesis H1, which suggests that the implementation of pilot free trade zones policy has the potential to compel enterprises to undergo a green transformation.

The green transformation of enterprises possesses the characteristic of being a public good and generates positive externalities. The private benefits derived from some activities are found to be lower in comparison to the corresponding social benefits. Additionally, the expenses associated with development tend to be higher, resulting in a significantly reduced level of enterprise benefits. Moreover, the insufficient availability of funding for innovation poses significant limits on the green development initiatives undertaken by enterprises. Enterprises have limitations in their funding structure due to the inherent characteristics of high risk, unclear returns, and extended cycles associated with research and development operations [37]. Enterprises face challenges in meeting the financial requirements of green innovation investment when relying exclusively on internal finances [38]. The process of enterprises undergoing a green transformation is inherently characterized by its reliance on capital and technology. This transformational behavior entails a lengthy return cycle and entails risks, which can potentially result in increased costs for enterprises seeking external financing [39]. Pilot free trade zones has the potential to mitigate financial limitations faced by enterprises. And the policies associated with free trade zones can ease financing constraints by enhancing innovation efficiency and attracting foreign direct investment [40]. The innovative strategies for negative list management, government function transformation, and trade and investment facilitation have substantially improved the functioning and productivity of businesses operating within the free trade zones, while promoting the convenience. The initial implementation of a series of financial liberalization measures has made the capital flow mechanism within the free trade zones more effective. Through the integration of traditional capital flow channels and international capital flow channels, financial openness has been strengthened, which has been achieved through the establishment of free trade zones. This integration has transformed free trade zones into significant reservoirs of capital, enabling the regulation of capital flow and offering ample external funding sources. These resources serve to alleviate financing constraints faced by enterprises [11]. The ongoing reduction of the negative list of foreign investment, in conjunction with the extension of the pilot free trade zones program, has significantly diminished barriers to foreign investment access. Consequently, this has facilitated more convenient pathways for international capital to invest in the Chinese market. The initial edition of the "Special Management Measures for Foreign Investment Access in the pilot free trade zones (Negative List)" in the Shanghai free trade zones consisted of 190 items in 2013, which has significantly reduced to 27 in 2022. External capital can alleviate the financing constraints faced by domestic enterprises in the host country through direct injection [41]. The financial capabilities of multinational enterprises in the international financial market enable efficient alleviation of corporate financing limitations through cooperation between domestic enterprises and foreign investors [42]. In contrast, foreign direct investment has the potential to address funding limitations faced by businesses and enhance the efficiency of financial resource allocation through the reduction of information asymmetry within the credit market. Based on information asymmetry, the possession of a foreign investment history has emerged as a discernible indicator for banks to assess the caliber of enterprises.

Pilot free trade zones policy has resulted in the development of discernible industrial clusters, which in turn have had a significant impact on the local region as well as the neighboring areas [43]. The radiating pathway is established by the interplay of the free trade zones, the central urban area, and the surrounding region, leading to a dynamic and cyclical process of resource, labor, and market accumulation. Free trade zones has the potential to facilitate the development of industrial clusters inside developing enterprises. Emerging enterprises have the potential to signify the future trajectory of enterprise development. In the free trade zones, the concentration of industries can alter the supply and demand dynamics of established industries, which fosters the development of new enterprises and facilitates the advancement of industrial structures [44]. Additionally, pilot free trade zones policy has the effect of facilitating the movement of production factors, including capital, skilled labor, and technology across national borders, which encourages the development of industrial agglomeration effects, specifically in terms of human capital and material capital, within the designated regions [32]. Industrial agglomeration has the potential to facilitate technical advancements and promote environmentally sustainable practices within enterprises, ultimately contributing to the improvement of their green transformation.

Therefore, we propose hypothesis H2: Industrial agglomeration and financing constraints may be the two channels through which pilot free trade zones policy affect the green transformation of enterprises.

Scholars’ investigations demonstrate that green subsidies have positive impact on green innovation. Subsidies can mitigate the financial risk associated with research and development (R&D) for enterprises, which also stimulating increased investment in innovation [45]. Subsidies also can incentivize enterprises to engage in green innovation to gain a competitive edge in the marketplace. The signaling theory posits that subsidies allocated to a specific domain are perceived as an endorsement of the domain’s potential for growth and assistance. And government’s emphasis on green and environmentally friendly products can stimulate competition among businesses and accelerate the development and promotion of green innovation technologies [46]. The green transformation is typically time-consuming, capital-intensive, and carries high-risks, which has little short-term return for enterprises [47]. Enterprises contribute to the widespread adoption of energy-saving through technologies [48]. What’s more, urban innovation can support the green transformation of industries by enhancing a connection between green technology and traditional industries. Government should promote the development of new energy sources, including hydropower, nuclear power, biofuels, tidal energy and optimizing the energy consumption structure [49]. Furthermore, an enhancement in energy consumption structure directly influences the reduction of carbon emission intensity [50], which can foster an environment conducive to the green transformation of enterprises.

Therefore, we propose hypothesis H3: urban innovation and green subsidies play a moderating role in the relationship between free trade zones and enterprises’ green transformation.

## 3. Model construction and variable description

### (1) Benchmark model

This paper uses the Difference-in-Difference (DID) method to evaluate the impact of pilot free trade zones policy on the green transformation of enterprises [51]. The research group consists of listed enterprises located in areas covered by pilot policies, while the reference group consists of listed companies located outside of free trade zones. There are differences in the time of the experimental group receiving the policy intervention, and the DID term is set as the interaction form of multiplying the dummy variable of the policy implementation time and the group dummy variable (treat × post). This paper uses the dummy variable DID to indicate whether the city is set up as a free trade zones, that is, if a city is set up as a free trade zones within the above time range, DID = 1, otherwise, DID = 0. Among them, if the city establishes a free trade zone in the first half of the current year, it is included in the current year; If the city establishes a free trade zone in the second half of the current year, it will be included in the following year. The benchmark model is as follows:

$${\mathrm{Greentrans}}_{\mathrm{ij}}=\alpha_{0}+\alpha_{1}DIDa_{\mathrm{ij}}+\alpha_{2}\mathrm{control}+\gamma *\mathrm{year}+\mu_{i}+\varepsilon_{\mathrm{ij}}$$ (1)

The enterprise is represented by i in Formula (1), the year is represented by j, the green transformation of the enterprises is represented by Greentrans, and the virtual variable of pilot free trade zones policy is represented by DID. The value in the control group is 0, while the value in the research group is 1. Control represents the relevant control variable, year represents the time fixed effect, *μ* represents individual fixed effects, *ε* represents a random interference item.

### (2) Description of variables

#### Explained variable

Drawing on the research [52], this study measures the green transformation of enterprises using textual information disclosed in annual reports. Compared with content analysis method, text analysis method applies computer natural language processing technology to accurately recognize unstructured text information under large samples, greatly reducing error rate and improving judgment consistency. There are two reasons for choosing the annual report of listed companies as the observation text. Firstly, the green transformation is a crucial strategic information for listed companies, which will be disclosed in the most widely accessible public annual report, which is consistent with the summarizing and guiding characteristics of the annual report information [52]. Secondly, the annual reports of listed companies are mandatory information disclosure, with strict format requirements and standardized wording, which will greatly improve the efficiency of keyword matching. Therefore, it is feasible to use the number of green transformation words in the annual reports of listed companies to measure their green transformation in this research. Five key factors, including green capabilities based on products and production processes, employee training and participation in environmental issues, green organizational capabilities across internal functions, formal environmental management systems and procedures, and strategic planning based on environmental issues, can effectively promote the green transformation of enterprises. And the formulation of sustainable development strategies by management is an important foundation for enterprises to shift from simply pursuing economic benefits to paying more attention to the negative externalities of production activities on the environment [53]. Based on this, enterprises carry out specific green practices, including changes in regulatory management models, employee environmental education and publicity, etc. At a deeper level, the green transformation of enterprises requires more innovation in green technology to produce green products, reduce total pollution emissions, and achieve improved enterprise performance and sustainable development. In summary, based on policy documents such as the "Twelfth Five Year Plan", "Environmental Protection Law", "Technical Guidelines for Enterprise Environmental Behavior Evaluation", "Green Manufacturing Standardization White Paper", and "Made in China 2025", and referring to relevant research, scholars selected 113 key words for enterprise green transformation from five aspects: promotion initiatives, strategic concepts, technological innovation, pollution control, and monitoring management [54]. We calculate the word frequency of green transformation using the frequency of each keyword in the annual reports of listed companies, the natural logarithm of that frequency plus one is applied to characterize the green transformation of companies.

#### Explanatory variable

There is a difference in the timing of policy intervention in the experimental group in this study, and the double difference is set as the interaction form of the policy implementation time dummy variable multiplied by the grouping dummy variable (treat × Post). This study uses the dummy variable DID to represent whether a city has been established as a free trade zone, that is, if a city has been established as a free trade zone within the aforementioned time range, DID = 1, otherwise DID = 0. Among them, if the city established a free trade zone in the first half of that year, it will be included in that year. If the city establishes a free trade zone in the second half of that year, it will be counted towards the following year.

#### Control variables

Regarding the selection of control variables, other factors in the production and operation activities of the enterprise can also have an impact on the green transformation of the enterprise. We select enterprise size (Size), monthly excess turnover rate (Dturn), management expense growth rate (Managexpenratio), sales expense growth rate (Salesgrowth), liquidity ratio (Liquidratio), and speed ratio (Speedratio) as control variables.

#### Mediating variables

Financial constraints and Herfindahl index (HHI) are selected as mediating variables. We accumulate the square of the ratio of the main business income of each company in the industry to the total main business income of the industry to obtain the Herfindahl Index (HHI).

#### Adjusting variables

Urban Innovation (Innovation) and Green Subsidy (Greensubsidy) were selected as adjusting variables, and Urban Innovation was calculated based on the 2017 China Urban and Industrial Innovation Power Report.

### (3) Data resource

The data is composed of panel data of A-share listed companies from 2009 to 2021, and all kinds of financial data of enterprises are from the database of CSMAR and the annual reports published by listed companies. In terms of macro data, the original data used in our study are mainly from China City Statistical Yearbook and various city statistical bulletins. Our study processed the data as follows: Excluding the annual samples of the financial industry, ST and PT companies and the samples of companies with missing data, and finally obtained 19,762 samples of A-share listed companies from 2009 to 2021.

## 4. Empirical analysis

### (1) Benchmark regression estimation

Table 1 shows the descriptive statistical results of the data of A-share listed companies from 2009 to 2021. It can be found from the table that the maximum value of green transformation of enterprises is 5.248, the minimum value is 1.352, and the mean is 3.171. From the perspective of the external environment of enterprises, the maximum value of innovation is 67.88, and the minimum value is 0. From the perspective of the company’s internal management, the maximum value of Dturn is 4.447, and the minimum value is -0.104. The maximum value of management expense growth rate is 26.96, and the minimum value is -0.863. Measuring the changes of enterprises from the perspectives of their external environment and internal management can more accurately judge the impact of pilot free trade zones policy on the green transformation of enterprises, laying a foundation for subsequent in-depth research.

**Table 1:** Descriptive statistics of variables.

Variables	Obs.	Means	Std.Dev	Min	Max
Greentrans	19,762	3.171	0.815	1.352	5.248
DID	19,762	0.248	0.432	0	1
Size	19,762	22.29	1.316	19.03	28.64
Dturn	19,762	-0.104	0.495	-6.886	4.447
Liquidratio	19,762	2.540	3.624	0.0385	190.9
Speedratio	19,762	2.025	3.347	0.0385	179.6
Salesgrowth	19,762	0.407	9.700	-1	902.7
Managexpenratio	19,762	0.160	0.610	-0.863	26.96
Financ	19,762	0.861	2.329	-11.34	12.05
HHI	19,618	0.210	0.184	0	1
Innovation	19,762	0.348	1.967	0	67.88
Greensubsidy	19,762	0.0118	0.0427	0	0.333

The benchmark regression results are shown in Table 2, where column (1) only shows the regression results of the free trade zones on the green transformation of enterprises. The regression coefficient of the free trade zones (DID) is significantly positive at the 1% level, which preliminarily indicates that pilot free trade zones policy is conducive to promoting the green transformation of enterprises. The regression coefficient of column (2), which includes both year and individual fixed effects and a series of control variables, including the free trade zones (DID), is significantly positive at the 1% level. This indicates that the hypothesis that pilot free trade zones policy can improve the green transformation of enterprises has a certain degree of credibility, which preliminarily verifies hypothesis H1. In terms of controlling variables, enterprise size (Size), monthly turnover rate (Dturn), sales expense growth rate (Salesgrowth), liquidity ratio (Liquidratio), and speed ratio have an impact on the green transformation of enterprises. Among them, sales expense growth rate, liquidity ratio (Liquidratio), and speed ratio have a significant impact on improving the green transformation of enterprises, indicating the importance of internal optimization management in the process of enhancing the green transformation of enterprises.

**Table 2:** Benchmark regression.

Variables	(1)	(2)
	Greentrans	Greentrans
DID	0.0376***	0.0366***
	(2.9996)	(2.8446)
Size		0.0155*
		(1.7415)
Dturn		-0.0187**
		(-2.4621)
Managexpenratio		0.0015
		(0.2682)
Salesgrowth		-0.0007***
		(-2.9889)
Liquidratio		0.0306***
		(3.1102)
Speedratio		-0.0319***
		(-3.0320)
Year fe	YES	YES
Individual fe	YES	YES
Constant	3.1617***	1.4346***
	(710.6920)	(7.4807)
Obs.	19758	19758
R^2^	0.6997	0.7000

### (2) Parallel trend test

**Fig 1:**
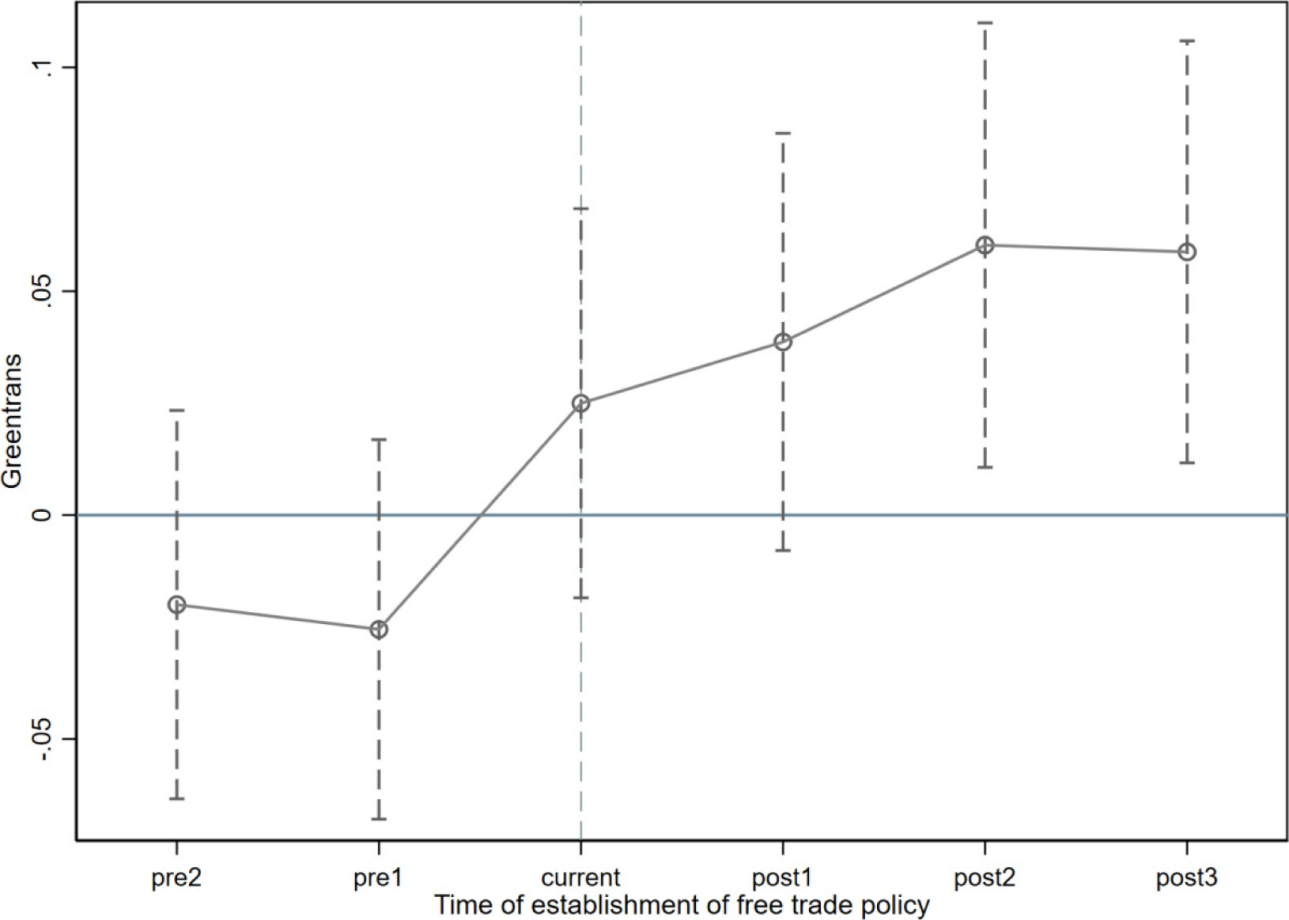
Parallel trend test.

As shown in Fig 1, this study uses the event study method to decompose the dynamic trend of economic effects of policies. When dealing with the policy time point, the current time minus the implementation time of the respective policy is used to determine the multi-phase DID. Based on this, we establish the following regression model:

$${\mathrm{Greentrans}}_{it}=\alpha +\sum_{j=-M}^{N}\varphi_{j}DID_{i,i-j}+\eta X_{it}+\delta_{i}+\gamma_{i}+\mu_{it}$$ (2)

GTFP_it_ indicates the green transformation of enterprises. DID_i, i-j_ is a dummy variable, indicating that enterprise i enjoyed the welfare policy of pilot free trade zones policy during the t-j period, and 1 is taken at this time, and 0 is taken otherwise. M and N in the model represent the number of periods before and after the policy time point. If φ-M to φ-1 are all significantly 0, it means that there is no significant difference between the treatment group and the control group in the first 1-M period before the implementation of the policy, that is, there is no significant difference between the innovation ability of the treatment group and the control group before pilot free trade zones policy, and the parallel trend test passes. In this model, the third year before the implementation of the policy is taken as the base period. As shown in the figure, the dashed line represents the confidence interval, and the estimated coefficient fluctuates around 0 before pilot free trade zones policy, while the estimated coefficient is not 0 in the years after pilot free trade zones policy, and remains positive as pilot free trade zones policy time increases. There was no significant difference in green transformation between the treatment group and the control group before pilot free trade zones policy, satisfying the parallel trend hypothesis. The positive policy effect on green transformation of enterprises after pilot free trade zones policy has been sustained within the research scope of this study, and the conclusion is robust.

### (3) Robustness test

#### 1. Placebo test

**Fig 2:**
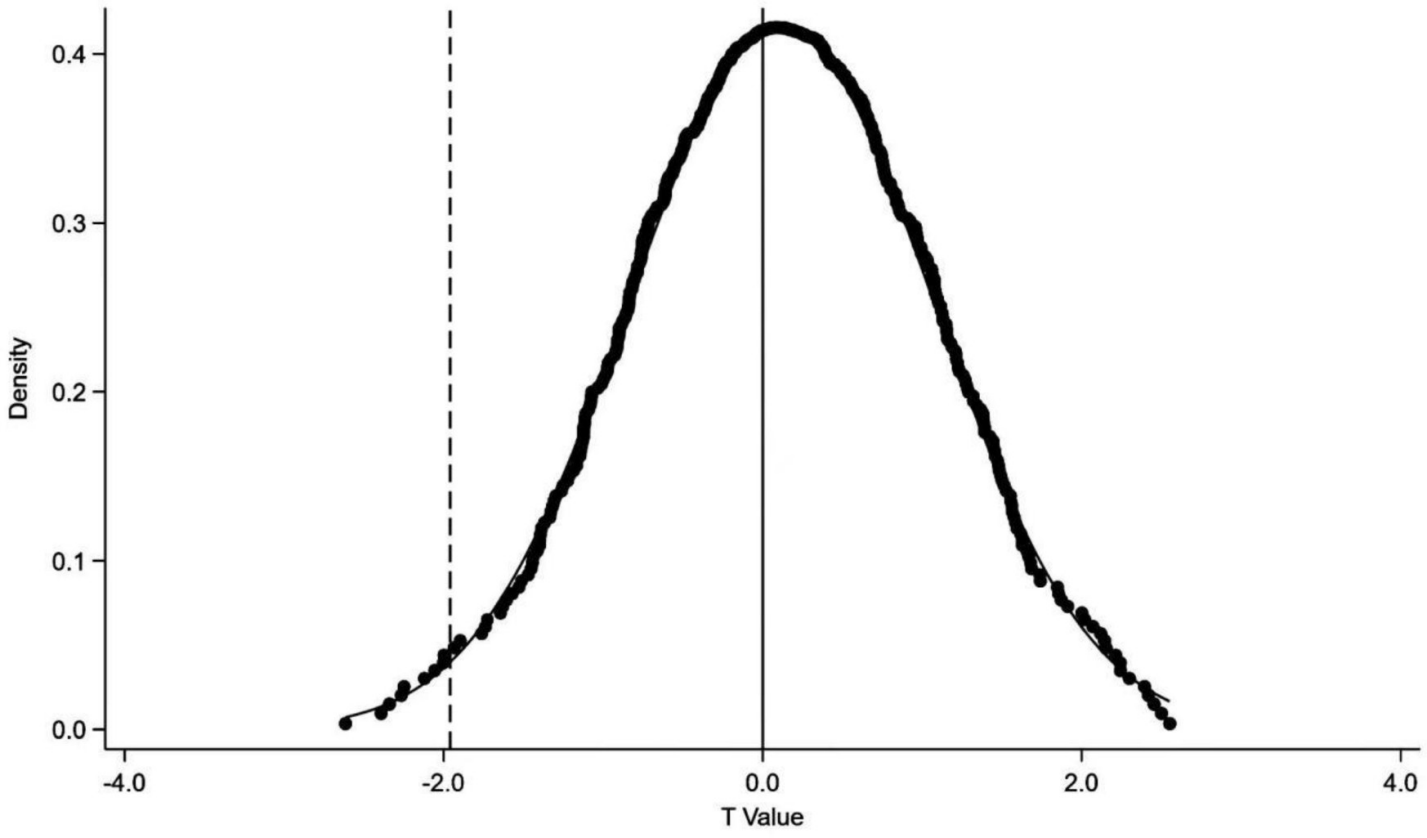
Placebo test diagram.

Our research addresses the issues by randomly selecting some cities as the virtual processing group, the number is equal to the number of cities where the original processing group free trade zones is located, randomly give a year for each city in the virtual processing group, as the time of the establishment of the free trade zones, repeat 500 times, get the estimated coefficient of 500 free trade zones (DID) and the corresponding T value. As shown in Fig 2, the estimated coefficient of the free trade zones (DID) remained near zero, and the T-value is mostly greater than 0.1, which is not significant. This shows that other uncontrollable or unobservable factors are difficult to promote the improvement of green transformation of enterprises, and the free trade zones are still the important factors to promote the improvement of green transformation of enterprises, which proves the robustness of the above conclusions.

#### 2. PSM-DID test

**Fig 3:**
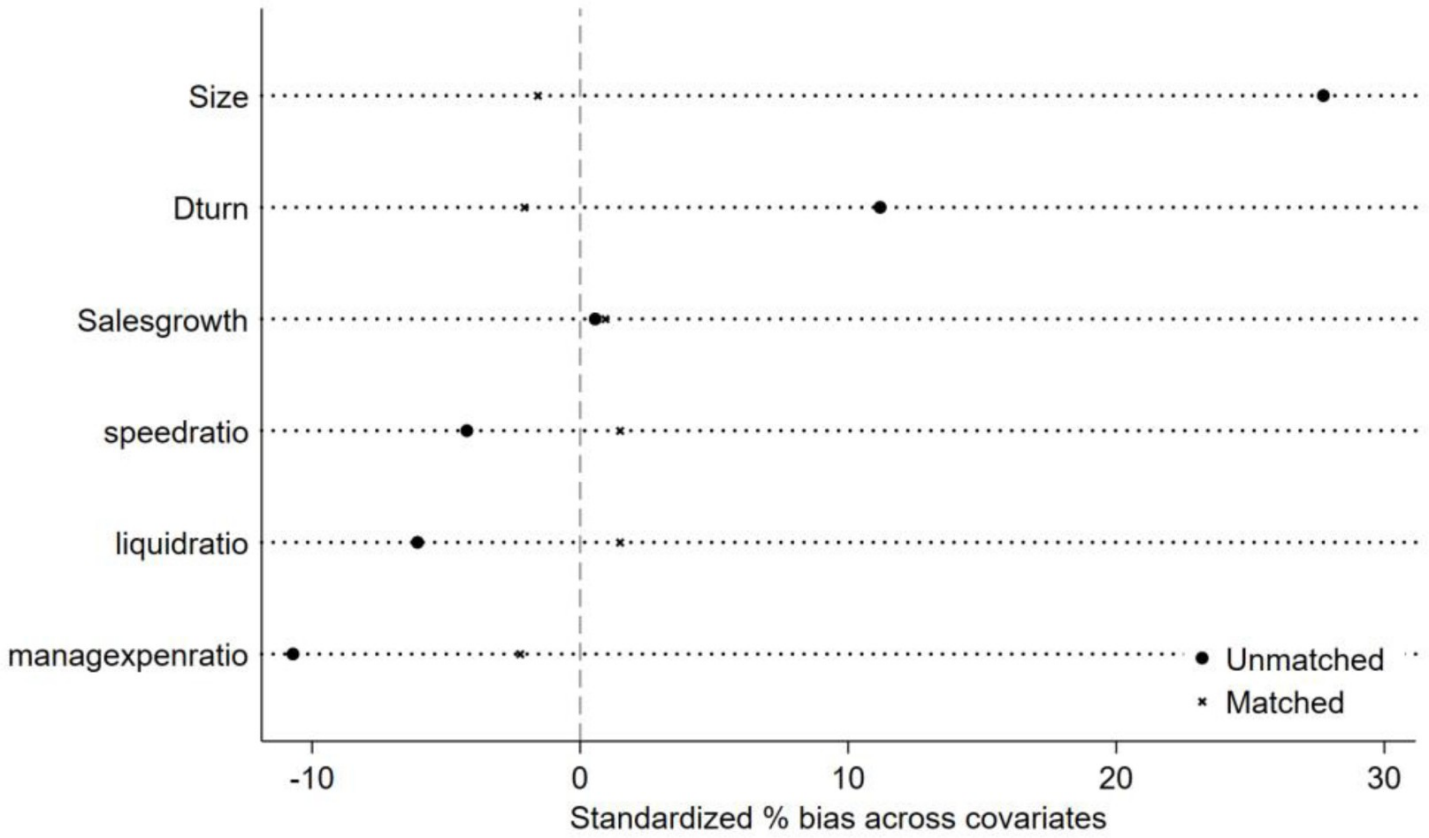
PSM-DID test.

In order to test the matching effect of propensity score, we conduct a balance test before and after matching for each continuous variable, to test whether there is a significant difference in the value of covariate between the two groups after matching. If the difference is not obvious, it indicates that the matching effect is good, and it is more appropriate to use such matching samples for DID regression. As shown in Fig 3, after matching, the standardization deviation is obviously reduced to near 0, indicating that the matching effect of main variables is accepted.

#### 3. Further robustness test

In our research, pilot free trade zones policy is lagged by one period for robustness test. As shown in columns (1) and (2) of Table 3, the estimated results of one-period-lagged pilot free trade zones policy are significantly positive, indicating that the impact on the green transformation of enterprises has inertia or path dependence. Compared with other cities, national central cities have more obvious advantages in political and economic status, which has a certain influence on the green transformation of enterprises. At the same time, in order to further verify the robustness of the above empirical results, we remove the sample size of four national central cities: Beijing, Shanghai, Chongqing and Tianjin. Columns (3) and (4) of Table 3 show that the results are still significant after reducing the sample size.

**Table 3:** Further robustness test results.

Variables	(1)	(2)	(3)	(4)
	Greentrans	Greentrans	Greentrans	Greentrans
L.DID	0.0344**	0.0321*		
	(2.1467)	(1.9442)		
DID			0.0445***	0.0437***
			(3.0149)	(2.8456)
Size		0.0171		0.0104
		(1.4732)		(1.0435)
Dturn		-0.0116		-0.0237***
		(-0.9192)		(-2.8418)
Managexpenratio		-0.0012		0.0053
		(-0.1646)		(0.9109)
Salesgrowth		-0.0009***		-0.0006**
		(-2.9862)		(-2.5402)
Liquidratio		0.0361***		0.0330***
		(2.7771)		(2.8944)
Speedratio		-0.0378***		-0.0348***
		(-2.6717)		(-2.8357)
Year fe	YES	YES	YES	YES
Individual fe	YES	YES	YES	YES
Constant	3.2451***	1.5980***	3.1614***	1.5426***
	(658.3152)	(6.2968)	(671.5907)	(7.2012)
Obs.	14987	14987	15807	15807
R^2^	0.6574	0.6576	0.6971	0.6973

#### (4) Mechanism test

Drawing upon theoretical analysis, we investigate the impact of pilot free trade zones policy on the green transformation of enterprises through the mediating effect of industrial agglomeration and financial constraints. The benchmark regression results indicate that the green transformation of enterprises is indeed positively affected by pilot free trade zones policy. To further verify the rationality of H2 and explore the specific transmission path of the impact, the model is established as follows:

$$Y=\mathrm{cX}+e_{1}$$ (3)

$$M=\mathrm{aX}+e_{2}$$ (4)

$$Y=c'X+\mathrm{bM}+e_{3}$$ (5)

The test logic is whether the regression coefficient c of X in Eq (3) is significant. If it is statistically significant, it proves that there is an intermediary effect. If the regression coefficient c is not significant, the follow-up test should be continued. Then, regression coefficient a of the dependent variable X in Eq (4) and regression coefficient b of the intermediary variable M in Eq (5) are tested. If both coefficients b and a are statistically significant, it indicates that the intermediary effect is significant. Finally, to test whether the regression coefficient c’ of the independent variable X in Eq (3) has statistical significance, if the coefficient c’ is not statistically significant, it means that the direct effect is not significant, and only the intermediary effect exists, that is, the complete intermediary effect.

The regression results in columns (1) and (2) of Table 4 show that the free trade zones policy has a significant impact on the two mechanism variables. Combined with the regression results, it can be concluded that pilot free trade zones policy can affect the green transformation of enterprises through industrial agglomeration and financial constraints. Following the establishment of the free trade zones, a favorable institutional environment facilitates the accumulation of superior human and physical capital in the zones. This allows for the cross-regional reallocation of capital, technology, and other production factors, ultimately optimizing the efficiency of resource allocation. The agglomeration of talents and enterprises will intensify the competition effect and spillover effect of innovation in the free trade zones, and enhance the technological innovation and regional innovation vitality of enterprises in the free trade zones [55], which will help enterprises absorb advanced technologies for green transformation. The institutional innovation and reform of the free trade zones have improved the power and vitality of technological innovation of enterprises, and the quantity and quality of patents have increased accordingly. As an important output form of enterprise innovation, patents are also technical assets of enterprises. Patents can be used as a signal to convey the quality of enterprises to investors, which can alleviate the information asymmetry between investors and enterprises, thus alleviating the financing constraints of enterprises [56]. Enterprises can also obtain bank discount loans, tax exemptions and government funding and subsidies through patent pledge, thus easing financing constraints [57], and thus solving the dilemma of green innovation transformation due to the lack of internal funds. Therefore, H2 is verified.

**Table 4:** Results of mechanism test.

Variables	(1)	(2)
	Greentrans	Greentrans
DID	0.7324***	0.7418***
	(60.6190)	(60.7743)
Financ	-0.0455***	
	(-18.8876)	
HHI		0.0926***
		(3.2586)
Size	0.0805***	0.0728***
	(19.4711)	(17.4168)
Dturn	0.0745***	0.0726***
	(7.0702)	(6.7885)
Managexpenratio	-0.1040***	-0.0939***
	(-11.5382)	(-10.3071)
Salesgrowth	0.0009	0.0007
	(1.5527)	(1.1846)
Liquidratio	-0.0147	-0.0362***
	(-1.5309)	(-3.7395)
Speedratio	0.0074	0.0414***
	(0.7066)	(3.9616)
Constant	1.2797***	1.3760***
	(13.7475)	(14.5995)
Obs.	19762	19618
R^2^	0.2095	0.1938

### (5) Heterogeneity test

Considering that the green transformation of enterprises needs technical support and whether the enterprises themselves are in polluting industries, we categorize enterprises into high-tech enterprises and non-high-tech enterprises, as well as heavy polluting enterprises and non-heavy polluting enterprises, and the regression tests are conducted separately.

The regression results can be seen in Columns (1) and (2) of Table 5, and the positive effect of pilot free trade zones policy on the green transformation of high-tech enterprises is more obvious. The regression coefficient of high-tech enterprises is 0.0460, which is significantly positive at the level of 1%, indicating that the establishment of the free trade zones has greatly alleviated the financing constraints of high-tech enterprises, while the impact on non-high-tech enterprises is not significant. Compared with non-high-tech enterprises, high-tech enterprises have greater advantages and volume in capital, talents and technology, which not only have diversified financing channels, but also can bear the cost of green transformation. Moreover, it enjoys the support of free trade zones in tax policies, high-tech enterprise policy subsidies and other aspects.

**Table 5:** Heterogeneity test results.

Variables	High-tech enterprises	Non-high-tech enterprises	Heavy polluting enterprises	Non-heavy polluting enterprises
	(1)	(2)	(3)	(4)
DID	0.0460**	0.0216	0.0467*	0.0335**
	(2.4214)	(1.1461)	(1.7333)	(2.2157)
Size	0.0008	0.0313**	0.0103	0.0125
	(0.0629)	(2.2892)	(0.5634)	(1.1544)
Dturn	-0.0235**	-0.0145	0.0036	-0.0277***
	(-2.2041)	(-1.2300)	(0.2375)	(-3.1354)
managexpenratio	-0.0018	-0.0018	0.0021	-0.0001
	(-0.2225)	(-0.2193)	(0.2268)	(-0.0168)
Salesgrowth	0.0046**	-0.0008***	-0.0002	-0.0008***
	(2.4377)	(-3.1808)	(-0.4116)	(-3.0333)
liquidratio	0.0444***	0.0205*	0.0181	0.0334***
	(2.9827)	(1.6930)	(0.8770)	(2.8836)
speedratio	-0.0452***	-0.0236*	-0.0173	-0.0354***
	(-2.8490)	(-1.7884)	(-0.7804)	(-2.8650)
Year fe	YES	YES	YES	YES
Individual fe	YES	YES	YES	YES
Constant	1.7335***	1.0980***	1.5387***	1.5039***
	(6.2590)	(3.6762)	(3.8786)	(6.4313)
Obs.	10765	8958	5814	13891
R^2^	0.6705	0.7079	0.7006	0.6986

In columns (3) and (4) in Table 5, we divide the heavily polluting enterprises and the non-heavily polluting enterprises into two groups, and conducts regression tests respectively. Pilot free trade zones policy has a more obvious positive effect on the green transformation of non-heavy polluting enterprises. And the regression coefficient of non-heavy polluting enterprises is 0.0335, which is significantly positive at the level of 1%. Compared with heavy polluting enterprises, the positive effect of pilot free trade zones policy on the green transformation of non-heavy polluting enterprises is more obvious. Heavy polluting enterprises are under the pressure of environmental regulation policies and the requirements of green emission reduction are higher than non-heavy polluting enterprises, so the cost of green transformation of heavy polluting enterprises is higher. Even if they enjoy the preferential policies of the free trade zones on the same basis as non-heavy polluting enterprises, the difficulty of green transformation of heavy polluting enterprises is higher than that of non-heavy polluting enterprises. What’s more, heavy polluting enterprises are subject to strict supervision by new media, which increases costs of green transformation in heavy polluting enterprises [58].

## 5. Further analysis

According to the previous theoretical analysis, we believe that under the influence of urban innovation and green subsidy, the impact especially the positive inpact of pilot free trade zones policy on enterprise green transformation will be enhanced. In our study, urban innovation (Innovation) and green subsidy (Greensubsidy) are used as moderator variables to cross-multiply with DID variable, and the interaction term of pilot free trade zones policy and urban innovation (DID ×Innovation), interaction term of pilot free trade zones policy and green subsidy (DID ×Greensubsidy). Specific models are set as follows:

$${\mathrm{Greentrans}}_{\mathrm{ij}}=\alpha_{0}+\alpha_{1}DIDa_{\mathrm{ij}}+\alpha_{2}DID*Innovation+\alpha_{3}\mathrm{control}+\gamma *\mathrm{year}+\mu_{i}+\varepsilon_{\mathrm{ij}}$$ (6)

$${\mathrm{Greentrans}}_{\mathrm{ij}}=\alpha_{0}+\alpha_{1}DIDa_{\mathrm{ij}}+\alpha_{2}DID*\mathrm{Greensubsidy}+\alpha_{3}\mathrm{control}+\gamma *\mathrm{year}+\mu_{i}+\varepsilon_{\mathrm{ij}}$$ (7)

Regression is conducted on Models (6) and (7) to explore the impact of interaction terms on corporate green transformation under the condition of heterogeneity. The results in Table 6 shows that the more perfect the urban innovation environment is, the better space and opportunities will be provided for the green transformation of enterprises. In the medium term of enterprise green transformation, it usually takes a long time, need large investment, involve high risk, and less return, which is unfavorable for enterprises with capital shortage and weak risk tolerance. The government’s green subsidies provide enterprises with additional financial support and reduce the financial risk of R&D activities, which makes enterprises more willing to increase R&D investment for technological innovation and accelerate the pace of green transformation. Thus, hypothesis H3 is verified.

**Table 6:** Regression results of adjustment effect.

Variables	(1)	(2)
	Greentrans	Greentrans
DID	0.0332**	0.0251*
	(2.5353)	(1.8973)
DIDxInnovation	0.0051*	
	(1.8380)	
DIDxGreensubsidy		1.6627***
		(7.2494)
Size	0.0155*	0.0172*
	(1.7426)	(1.9282)
Dturn	-0.0187**	-0.0186**
	(-2.3668)	(-2.3587)
Managexpenratio	0.0015	0.0010
	(0.2774)	(0.1818)
Salesgrowth	-0.0007***	-0.0007***
	(-3.6797)	(-3.6659)
Liquidratio	0.0305***	0.0310***
	(3.2101)	(3.2490)
Speedratio	-0.0317***	-0.0324***
	(-3.1322)	(-3.1800)
Year fe	YES	YES
Individual fe	YES	YES
Constant	1.4335***	1.3998***
	(7.4741)	(7.3022)
Obs.	19762	19762
R^2^	0.6870	0.6875

## 6. Conclusion

Using the micro data of China’s listed enterprises from 2009 to 2021, this study discusses the impact of pilot free trade zones policy on the green transformation of enterprises, and empirically tests the impact of the free trade zones on the green transformation of enterprises. The main conclusions are as follows: first, pilot free trade zones policy can significantly improve the green transformation of enterprises in the zones. Second, free trade zones have an impact on enterprises’ green transformation through industrial agglomeration and financial constraints. Third, the heterogeneity test analysis implies that the impact of the free trade zones on the green transformation of enterprises vary according to the industry in which the enterprises are located. Fourth, urban innovation and green subsidies play a positive moderating role in the impact of free trade zones on enterprises’ green transformation. According to the conclusions, this paper proposes the following policy implications.

Firstly, fully utilizing the inherent institutional innovation capability of the free trade zones is conducive to improving the investment environment. Especially in terms of foreign investment access, it is crucial to further streamline the negative list, thereby increasing capital inflows, which will increase foreign investment in advanced green transformation.

Secondly, effectively allocating a large amount of financial resources is crucial for developing innovative technologies and products. It is urgent for government entities to consistently evaluate the effectiveness of subsidy policies centered on research and development in order to maintain their effectiveness. In order suitable for enterprises with different types of property rights, the government can formulate subsidy policies for each specific category.

Thirdly, further improving the admission standards and business environments to attract high-tech foreign enterprises, and guiding the investment into the high-tech and green industries is vital for the government. More positive environment creation drives domestic enterprises to achieve more efficient green transformation.

Lastly, deeply recognizing the importance of subsidies for the enterprises’ green transformation is very important for the government. Therefore a comprehensive and multi-level subsidy system should be established for the research, development, and green technology implementation. At the same time, monitoring the changes in the market and industry in order to adjust subsidy policies promtly is also crucial for the government. For example, the government should increase subsidies for heavy polluting enterprises and non high-tech enterprises to alleviate the green transformation costs.

There are some limitations of this paper. In this research, we empirically test that industrial agglomeration and financial constraints are the driving force of pilot free trade zones policy to promote green transformation of enterprises. However, the industrial agglomeration greatly affected by the competition effect, and this needs to be considered in the future research. In addition, with increasing numbers of free trade zones, different characteristics have been emerged. However, due to the length of time, it is difficult to compare the policy effects in different free trade zones, this is our future research concern.
